# A Cluster-Randomised Intervention Trial against *Schistosoma japonicum* in the Peoples' Republic of China: Bovine and Human Transmission

**DOI:** 10.1371/journal.pone.0005900

**Published:** 2009-06-12

**Authors:** Darren J. Gray, Gail M. Williams, Yuesheng Li, Honggen Chen, Simon J. Forsyth, Robert S. Li, Adrian G. Barnett, Jiagang Guo, Allen G. Ross, Zheng Feng, Donald P. McManus

**Affiliations:** 1 Molecular Parasitology Laboratory, Queensland Institute of Medical Research, Brisbane, Queensland, Australia; 2 School of Population Health, The University of Queensland, Brisbane, Australia; 3 Hunan Institute of Parasitic Diseases, WHO Collaborating Centre for Research and Control on Schistosomiasis in Lake Region, Huabanqiao Road, Yueyang, People's Republic of China; 4 Jiangxi Provincial Institute of Parasitic Diseases, Nanchang, People's Republic of China; 5 School of Public Health, Queensland University of Technology, Brisbane, Queensland, Australia; 6 National Institute of Parasitic Diseases, Chinese Centre for Disease Control and Prevention, Shanghai, People's Republic of China; 7 School of Public Health, Griffith University, Meadowbrook, Queensland, Australia; University of California Los Angeles, United States of America

## Abstract

**Background:**

Zoonotic schistosomiasis japonica is a major public health problem in China. Bovines, particularly water buffaloes, are thought to play a major role in the transmission of schistosomiasis to humans in China. Preliminary results (1998–2003) of a praziquantel (PZQ)-based pilot intervention study we undertook provided proof of principle that water buffaloes are major reservoir hosts for *S. japonicum* in the Poyang Lake region, Jiangxi Province.

**Methods and Findings:**

Here we present the results of a cluster-randomised intervention trial (2004–2007) undertaken in Hunan and Jiangxi Provinces, with increased power and more general applicability to the lake and marshlands regions of southern China. The trial involved four matched pairs of villages with one village within each pair randomly selected as a control (human PZQ treatment only), leaving the other as the intervention (human and bovine PZQ treatment). A sentinel cohort of people to be monitored for new infections for the duration of the study was selected from each village. Results showed that combined human and bovine chemotherapy with PZQ had a greater effect on human incidence than human PZQ treatment alone.

**Conclusions:**

The results from this study, supported by previous experimental evidence, confirms that bovines are the major reservoir host of human schistosomiasis in the lake and marshland regions of southern China, and reinforce the rationale for the development and deployment of a transmission blocking anti-*S. japonicum* vaccine targeting bovines.

**Trial Registration:**

Australian New Zealand Clinical Trials Registry ACTRN12609000263291

## Introduction

Schistosomiasis is a major public health concern in China with approximately one million people infected and 59 million at risk of infection [1]–[4]. The majority (>80%) of cases occur around the lake (Dongting and Poyang) and marshland regions of five provinces in southern China - Hunan, Jiangxi, Anhui, Hubei and Jiangsu. Transmission of *Schistosoma japonicum* also occurs in the mountainous regions of Sichuan and Yunnan provinces [1], [4]–[8].

Unlike African schistosomiasis, schistosomiasis japonica is a zoonosis, with over 40 species of wild and domestic animals, comprising 28 genera and 7 orders, able to harbour the infection [9]. The range of mammalian hosts complicates control efforts and the economic burden associated with schistosomiasis morbidity and mortality has taken its toll on both human and livestock populations surrounding the lake regions [1].

There is substantial evidence indicating that bovines, particularly water buffaloes (*Bubalus bubalis*), play a major role in the transmission of *S. japonicum* to humans in China [1], [2], [4], [9]–[21]. The daily faecal output from a water buffalo (∼25 kg) has been estimated to be at least 100 times that (250 g) produced by a human individual [1], [11]. Accordingly, a recent study has shown that the environmental contamination attributable to 238 infected bovines (225/13; water buffaloes/cattle) was, in total, approximately 28.7 million eggs/day [18], emphasizing their considerable contribution in the deposition of *S. japonicum* eggs into the external environment. Moreover, a praziquantel (PZQ)-based pilot intervention study we undertook (1998–2003) [15] provided proof of principle that water buffaloes are major reservoir hosts for *S. japonicum* around the Poyang Lake region of Jiangxi Province [15]. Mathematical modelling [22] supported this conclusion and predicted that these bovines are responsible for approximately 75% of human transmission in this setting [15]. Further support came from a molecular field survey of *S. japonicum* in China using microsatellite markers, which showed that humans and bovines contribute considerably more to the parasite reservoir than other definitive host species [23].

Our pilot intervention study [15] provided the first definitive experimental evidence that water buffaloes are important reservoirs for *S. japonicum* transmission in China. Building on this achievement, we present here the results of a more stringent bovine intervention trial (2004–2007) we undertook using a cluster-randomised design with increased power and with more general applicability to the lake and marshland regions of southern China.

## Methods

The protocol for this trial and supporting CONSORT checklist are available as supporting information; see Checklist S1 and Protocol S1.

### Study Design

The study design, study areas and baseline findings have been described elsewhere [18]. In brief, we carried out a cluster-randomised intervention trial, which involved four matched village pairs in Hunan and Jiangxi provinces. One village in each pair was randomly selected as an intervention village (human and bovine praziquantel treatment) and the remaining village in the pair served as a control village (human praziquantel treatment only). A sentinel cohort of people, to be monitored for new infections for the duration of the study was selected from each village. The primary end point of the trial was human infection incidences. Other outcome measures included: human infection intensity, bovine infection rates and intensity of infection.

The timing of bovine mass treatment was changed slightly from that originally intended (March/April) [18] to more closely coincide with the time between transmission periods. It was actually undertaken between May and August, the exact timing dependent on the variable yearly rainy season.

### Faecal examinations

Two stool samples per person were examined microscopically using the Kato-Katz thick smear technique [24], with three slides (read blind) per stool, to determine *S. japonicum* infection rates and intensity of infection. Bovine stool samples were examined for *S. japonicum* infection rates using the miracidial hatching test (3 individual hatchings read blind (50 grams faeces/hatching)) and intensity of infection, using a traditional Chinese sedimentation method [16].

### Study sites

The originally selected village pair of Aigou and Dingshan (Jiangxi Province) was replaced after baseline (2004) by an alternative village pair (Cao Jia and Yufeng) selected [18] with similar characteristics (Table S1) to the original villages. The baseline survey of Cao Jia and Yufeng was undertaken in 2005, so this pair had one year less of follow-up than the others.

### Statistical analysis

Statistical analysis of study end points was similar to published procedures [15], [18] and was performed within the SAS programme [25]. Each cohort member was assigned a water contact score for each year preceding infection status assessment. This was determined by adding season-specific sub-scores based on frequency of water contact obtained through the yearly water contact surveys [18]. Poisson regression was used for formal analyses of human infection rates, both crude and adjusted (for water contact, using the water contact score). Clustering was accounted for by analysing the combined intervention effect within the matched pairs. GEE was used to account for correlations due to repeated measures. Snail infections were analysed by calculating prevalence and the density of infected snails per 100 metres-squared. The efficacy of bovine treatment in reducing human infection was calculated using the formula: Efficacy = 1−RR; where RR is the human relative risk.

### Ethical considerations

Written ethical approval for this study was obtained from the national, provincial and village levels within China, and the Human Research Ethics Committee of the Queensland Institute of Medical Research also granted approval for the study. Written informed consent was obtained from all adults and from parents or guardians of minors who were involved in the project. Study participants identified as stool egg-positive for schistosomiasis were treated with 40 mg/kg of praziquantel [26].

## Results

### Baseline

Baseline results have been previously reported [18]. For the replacement villages, baseline human and bovine prevalence (%) and intensity of infection (geometric mean eggs per gram (EPG) in infected individuals) for *S. japonicum* are shown in Tables 1 and 2. Human prevalence was marginally higher in Yufeng (control) compared to Cao Jia (intervention) (Table 1); while bovine prevalence was slightly higher in Cao Jia (intervention) compared to Yufeng (Control) (Table 2). The snail prevalence (%) and density of infected snails per 100 m^2^ at baseline were 0.85% and 1.21 per 100 m^2^ and 0.21% and 1.14 per 100 m^2^ for Yufeng and Cao Jia, respectively.

**Table 1:** Human infection rates (* replacement village pair).

Province	Hunan				Jiangxi			
Pair	Pair 1		Pair 2		Pair 3		Pair 4^*^	
Village Status	Control	Intervention	Control	Intervention	Control	Intervention	Control	Intervention
Administrative Village	Yongxiang	Mengjiang	Jizhong	Yongfu	Fuqian	Xindong	Yu Feng	Cao Jia
**Baseline**								
Sentinel Cohort #	363	335	467	334	671	751	415	441
Prevalence	7.7% (5.0–10.5)	8.7% (5.6–11.7)	13.9% (10.8–17.1)	18.9% (14.6–23.1)	11.9% (9.5–14.4)	13.8% (11.4–16.3)	19.8% (16.0–23.7)	13.2% (10.0–16.4)
Geometric Mean EPG in Infected	7.2 (5.6–9.2)	8.1 (6.1–10.8)	9.1 (7.2–11.4)	13.3 (9.8–18.0)	10.8 (8.6–13.6)	27.4 (20.3–36.9)	37.7 (26.6–53.3)	22.4 (16.0–31.4)
**Follow-up 1 Yr**								
Sentinel Cohort #	296	273	336	276	623	705	295	360
Incidence	4.4% (2.0–6.7)	6.2% (3.3–9.1)	6.8% (4.1–9.6)	9.4% (6.0–12.9)	11.4% (8.9–13.9)	9.8% (7.6–12.0)	23.4% (18.5–28.2)	1.9% (0.5–3.4)
Geometric Mean EPG in Infected	8.9 (5.9–13.4)	12.1 (6.9–21.1)	12.1 (6.8–21.4)	14.2 (7.8–25.8)	6.8 (5.8–7.9)	46.0 (33.8–62.4)	24.5 (16.9–35.6)	14.0 (7.1–27.6)
**Follow-up 2 Yrs**								
Sentinel Cohort #	270	239	282	226	575	639	216	294
Incidence	1.9% (0.2–3.5)	1.7% (0.0–3.3)	2.5% (0.7–4.3)	1.3% (0.0–2.8)	8.9% (6.5–11.2)	7.4% (5.3–9.4)	18.1% (12.9–23.2)	2.0 (0.4–3.7)
Geometric Mean EPG in Infected	5.5 (3.4–8.8)	6.5 (2.7–15.5)	22.9 (2.7–197.2)	6.0 (1.2–29.0)	10.1 (8.0–12.8)	47.0 (28.0–79.1)	13.2 (9.8–17.9)	13.8 (5.0–37.7)
**Follow-up 3 Yrs**								
Sentinel Cohort #	208	200	260	195	538	603		
Incidence	0.5% (0.0–1.4)	0.5% (0.0–1.5)	2.7% (0.7–4.7)	1.5% (0.0–3.3)	10.6% (8.0–13.2)	5.5% (3.7–7.3)		
Geometric Mean EPG in Infected	8.3 (N/A)	4.2 (N/A)	4.6 (3.6–5.9)	6.0 (1.2–29.0)	13.5 (10.4–17.4)	48.6 (28.9–82.0)		

**Table 2:** Bovine infection rates and treatment coverage (* replacement village pair).

Province	Hunan				Jiangxi			
Pair	Pair 1		Pair 2		Pair 3		Pair 4^*^	
Village Status	Control	Intervention	Control	Intervention	Control	Intervention	Control	Intervention
Administrative Village	Yongxiang	Mengjiang	Jizhong	Yongfu	Fuqian	Xindong	Yu Feng	Cao Jia
**Baseline**								
n	63	59	82	88	233	230	108	59
Prevalence	25.4% (14.3–36.4)	28.8% (16.9–40.7)	29.3% (19.2–39.3)	18.2% (10.0–26.4)	15.9% (11.2–20.6)	12.2% (7.9–16.4)	10.2% (4.4–16.0)	13.6% (4.6–22.6)
Geometric Mean EPG in Infected	4.9 (2.6–9.3)	5.5 (2.6–11.7)	7.2 (5–10.4)	3.4 (1.8–6.7)	1.9 (1.4–2.5)	0.8 (0.45–1.5)	2.1 (1.3–3.4)	0.5 (0.4–0.6)
Tx Coverage	38.2%	100%	33%	100%	0%	100%	0%	100%
**Follow-up 1 Yr**								
n	54	96	64	92	253	311	93	62
Infection Rate	25.9% (13.9–38.0)	17.2% (7.7–26.7)	25.0% (14.1–35.9)	15.2% (7.7–22.7)	13.4% (9.2–17.7)	9.3% (6.1–12.6)	14.0% (6.8–21.2)	4.8% (0.0–10.3)
Geometric Mean EPG in Infected	1.9 (1.2–2.9)	4.0 (1.8–8.7)	3.3 (1.9–5.9)	4.2 (2.2–8.1)	1.0 (0.7–1.3)	0.2 (0.2–0.3)	0.8 (0.5–1.4)	0.2 (N/A)
Tx Coverage	37.5%	90.7%	53.8%	100%	0%	100%	0%	100%
**Follow-up 2 Yrs**								
n	57	68	131	136	234	374	124	34
Infection Rate	26.3% (14.5–38.1)	17.6% (8.4–26.9)	24.4% (17.0–31.9)	11.8% (6.3–17.2)	10.7% (6.7–14.7)	1.6% (0.3–2.9)	12.9% (6.9–18.9)	2.9% (0.0–8.9)
Geometric Mean EPG in Infected	1.6 (0.9–2.9)	N/A	2.0 (1.4–2.9)	1.5 (1.0–2.2)	1.9 (1.6–2.4)	0.1 (0.1–0.2)	1.4 (0.9–2.0)	0.0 (N/A)
Tx Coverage	0%	93.4%	0%	92.1%	0%	100%	0%	100%
**Follow-up 3 Yrs**								
n	46	61	114	127	195	436		
Infection Rate	28.3% (14.7–41.8)	16.4% (6.8–25.6)	18.4% (11.2–25.6)	11.0% (5.5–16.5)	12.3% (7.7–17.0)	0.5% (0.0–1.1)		
Geometric Mean EPG in Infected	1.3 (0.5–3.4)	1.6 (0.9–3.0)	1.6 (1.1–2.3)	1.2 (0.8–1.7)	2.3 (1.8–2.8)	0.1 (N/A )		
Tx Coverage	0%	94.2%	0%	76.9%	0%	100%		

### Participant flow

**Figure 1:**
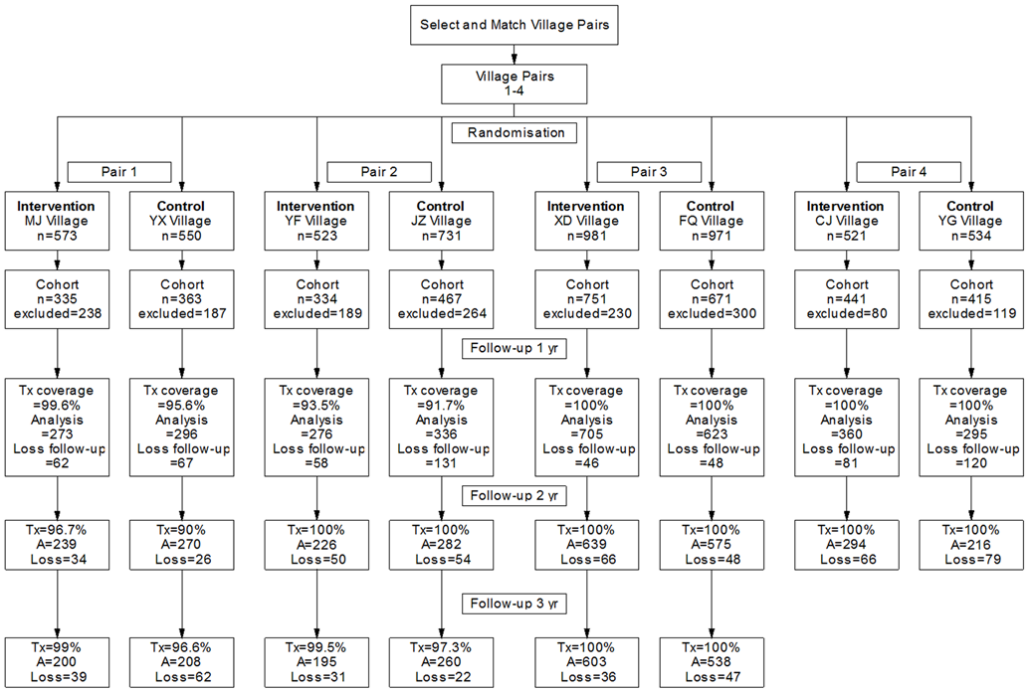
Trial profile.

Within each village a sentinel cohort of people was selected for follow-up over the course of the trial. The flow of these study participants is shown in Figure 1. Loss to follow-up per year ranged from 5.6% to 28.9%, with the majority of villages having an attrition rate of less than 20% per year as indicated in the original study design [8]. Jizhong had an attrition rate of 28.1% in the first year, which fell in subsequent years to 16.1% and 7.8%. Yufeng also had high attrition rates of 28.9% and 26.8%, although the cohort selected was higher than original design requirements [18].

Human treatment coverage was high, ranging generally from 90–100% except for Jizhong and Xindong, which had coverages of 89.7% and 87.6%, respectively, following baseline (Figure 1). Bovine treatment coverage in the intervention villages also ranged from 90–100% except for Yongfu, which had 76.9% in the 3^rd^ year of follow-up. Contrary to the study design, local farmers treated 33–53.8% of bovines in the Hunan province control villages following baseline and after one year of follow-up (Table 2).

### Follow-up

#### Human infection

Over the three years of follow-up, all of the Hunan province villages (pairs 1 & 2) had decreases in incidence and in the final year all incidences were low, ranging from 0.5% to 2.7% (Table 1). Although incidences were similar in the final year, the absolute reductions within the villages were not. Larger reductions in incidence were observed in the intervention villages compared with the control villages, within each matched pair, being 3.9% and 5.7%, respectively, in Yongxiang and Mengjiang, and 4.1% and 7.9% in Jizhong and Yongfu, respectively.

Upon conclusion of the trial in Jiangxi province (village pairs 3 & 4; 3 years follow-up Fuqian and Xindong; 2 years follow-up Yufeng and Cao Jia), the incidences were lower in the intervention villages compared with the controls, being 10.6% (95% CI 8.0–13.2) vs 5.5% (95% CI 3.7–7.3) in Fuqian and Xindong respectively; and 18.1% (95% CI 12.9–23.2) vs 2.0% (95% CI 0.4–3.7) in Yufeng and Cao Jia respectively (Table 1).

**Figure 2:**
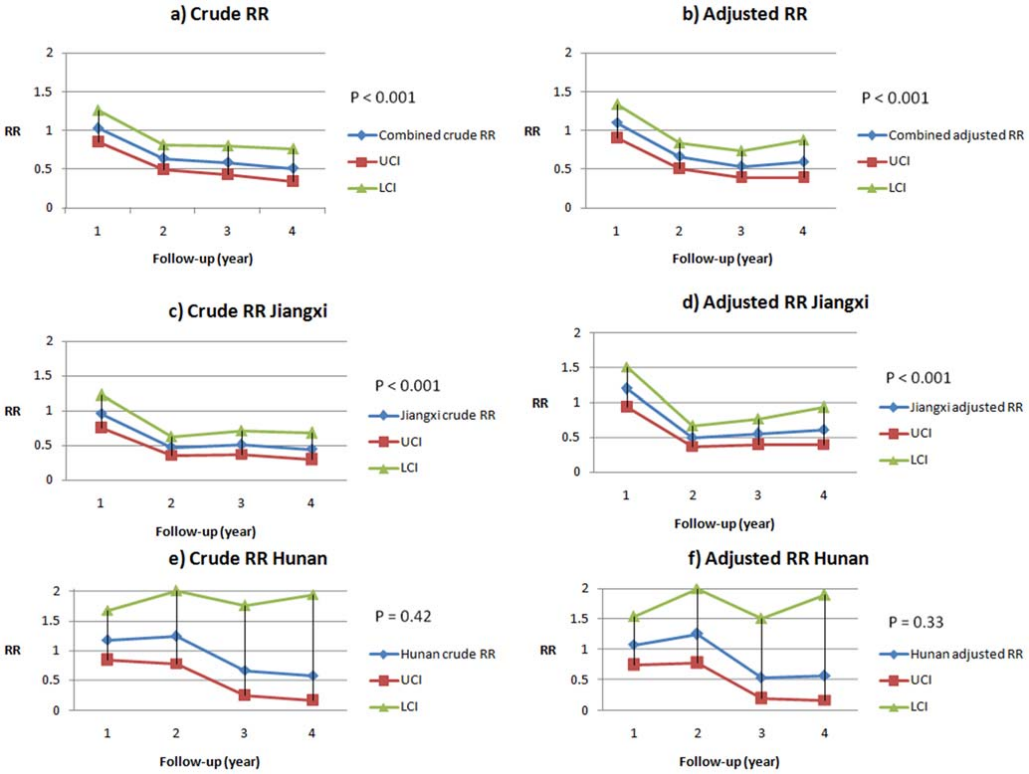
Yearly relative risks. Relative risks for each year of follow-up for Hunan province, Jiangxi province, and both provinces combined.

Poisson regression analyses, yielding crude and adjusted relative risks for each year of the trial within each province and for the entire trial (provinces combined), are shown in Figure 2. A downward trend was observed within both provinces individually and combined for both crude and adjusted relative risks, although there was a slight increase in the adjusted relative risk in the final year of follow-up. These results were significant overall (provinces combined) (P<0.001) and for Jiangxi (P<0.001) but not for Hunan (P = 0.42 (crude) and P = 0.32 (adjusted)), as indicated by the wide confidence intervals (Figure 2).

**Figure 3:**
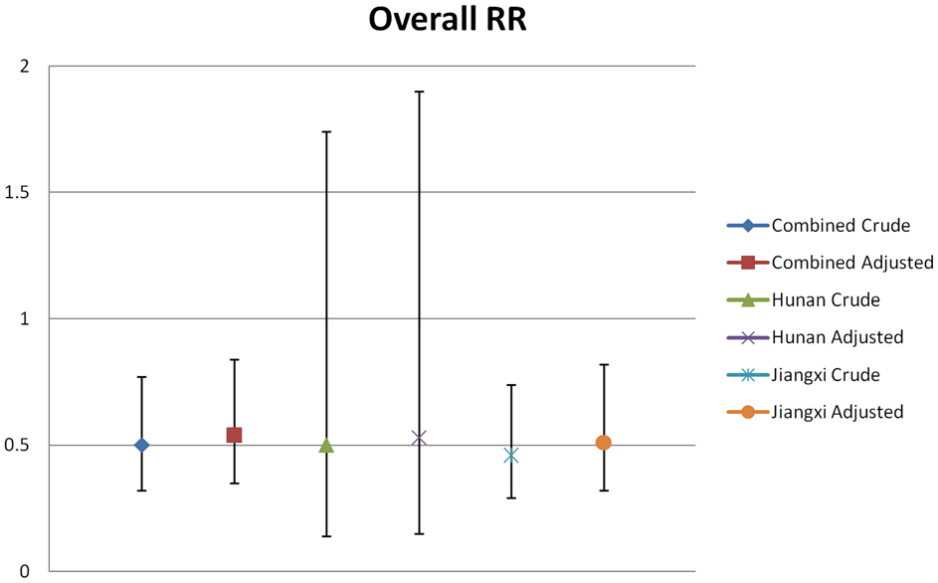
Overall relative risks. Relative risks for all years of follow-up for Hunan province, Jiangxi province, and both provinces combined.

Crude and adjusted relative risks for all years of follow-up within both provinces are shown in Figure 3. The adjusted relative risk for Hunan was RR = 0.53 (P = 0.32) and RR = 0.51 (P<0.001) for Jiangxi. Further regression analyses comparing the provincial relative risks showed that there were no differences between the two (P>0.05). Poisson regression analysis of both provinces combined yielded crude and adjusted relative risks of RR = 0.5 (P<0.001) and RR = 0.54 (P<0.001) (Figure 3). The efficacy (based on adjusted relative risks) of bovine PZQ chemotherapy for reducing human *S. japonicum* infection was calculated to be 47% in Hunan province, 49% in Jiangxi province, and 46% overall.

#### Bovine infection

Upon conclusion of the trial, the infection rates were lower in the intervention villages compared with the control village, 28.3% (95% CI 14.7–41.8) vs 16.4% (95% CI 6.8–25.6) for Yongxiang and Mengjiang; 18.4% (95% CI 11.2–25.6) vs 11% (95% CI 5.5–16.5) for Jizhong and Yongfu; 12.3% (95% CI 7.7–17.0) vs 0.5% (95% CI 0.0–1.1) for Fuqian and Xindong; and 12.9% (95% CI 6.9–18.9) vs 2.9% (95% CI 0.0–8.9) for Yu Feng and Cao Jia (Table 2). Larger reductions in the infection rates were, however, observed in the Jiangxi province intervention villages compared with those in Hunan province (Table 2).

#### Snail prevalence and density of infected snails

The prevalence and density of infected snails fluctuated substantially across the study villages and over the study period. Prevalence and density of infected snails was reduced to zero in Yongxiang village in 2006 due to environmental modification of the marshland within the village by the Chinese authorities.

## Discussion

This cluster-randomised intervention trial was carried out (2004–2007) in order to support the accumulating evidence that bovines are responsible for the majority of human *Schistosoma japonicum* transmission in the lake and marshland regions of southern China. The trial was designed to allow for the comparison of control (human treatment) and intervention (human and bovine treatment) villages within matched pairs, so as to determine the impact of bovine chemotherapy on human incidence. One of the originally selected village pairs, Aigou and Dingshan in Jiangxi Province, had to be replaced after the baseline data collection in 2004, due to the inclusion of the former village in a pilot initiative, whereby water buffaloes were supplanted by tractors as a new schistosomiasis control option [27]. Characteristics of the alternative village pair selected (Cao Jia and Yufeng), were similar, indicating our success in carefully matching the villages and subsequently reducing confounding (Table S1). Moreover, baseline survey results of the replacement pair, undertaken in 2005 were similar to the original pair. The delayed start of the trial resulted in there being only two years of follow-up in the replacement pair.

Trial results showed that combined human and bovine chemotherapy with PZQ had a greater effect on human incidence than human PZQ treatment alone. This is illustrated in Hunan province with the greater reduction in human *S. japonicum* incidence in the intervention compared to the control villages (Table 1); and in Jiangxi province with the lower human *S. japonicum* incidences in the intervention villages compared to their respective controls (Table 1).

This is reinforced by the reduction in the bovine infection rates within the intervention villages compared to minimal or no reductions in the control villages (Table 2). The snail infection results were inconclusive due to the fluctuations across the study villages over the course of the trial that, were a result of high levels of snail sampling variability due to spatial aggregation effects. However, we conclude that the reduction in the numbers of infected bovines within the intervention villages had an indirect effect on human incidence as seen in our earlier pilot drug intervention trial [15]. These findings are supported by Poisson regression analyses for all years of follow-up (Figure 3) that yielded adjusted relative risks of 0.53 (P = 0.32) and 0.51 (P = <0.001) for Hunan and Jiangxi provinces, respectively.

The lack of significance in the result for Hunan province can be attributable to several factors that likely diluted the intervention effect: a) the contamination of the control villages through the treatment of bovines by local farmers following the baseline and after 1 year of follow-up (Table 2); b) low bovine treatment coverage in the intervention villages compared to that observed in Jiangxi province (e.g. 76.9% Yongfu village in the 3^rd^ year of follow-up) (Table 2); and c) environmental modification—initiated by the Chinese Department of Agriculture—of the marshland area in Yongxiang village (control) in 2006, resulting in the removal of snails from the transmission cycle and the subsequent reduction in human infection rates (Table 1). These factors highlight some of the challenges faced when undertaking long-term longitudinal field trials of this type.

Further regression analyses comparing the relative risks of the two provinces showed that they were not significantly different, thus allowing subsequent Poisson regression analyses combining the two provinces yielding crude and adjusted relative risks of 0.5 (P = <0.001) and 0.54 (P = <0.001) (Figure 3). These results provide experimental proof that the incidence of human *S. japonicum* infection can be reduced through the reduction in infection rates in water buffaloes, thereby emphasising the important role of bovines (particularly water buffaloes) in human schistosomiasis transmission. Furthermore, the efficacy of twice-annual bovine PZQ chemotherapy for reducing human *S. japonicum* infection in the lake and marshland region of southern China was calculated to be 46%.

Interventions including bovine chemotherapy are likely to reduce the economic burden of schistosomiasis in China through not only the reduction in bovine infection, which affects agricultural productivity [1] but also by the reduction in human infection. It has been estimated that a loss in work productivity ranging from 16–88% in infected individuals (depending on intensity of infection) is a direct result of the morbidity associated with schistosomiasis [28]. Therefore, a 46% reduction in human infection would substantially increase the agricultural and economic productivity of these rural lakeside residents.

### Conclusions

The results from this study supported by previous experimental evidence [29], confirms that bovines are the major reservoir host of human schistosomiasis in the lake and marshland regions of southern China, and reinforce the rationale for the development and deployment of a transmission blocking anti-*S. japonicum* vaccine targeting bovines [29], [30], [31]. Furthermore, the study has shown that combining human and bovine chemotherapy is potentially an effective intervention for schistosomiasis control in this setting. However, it is labour intensive and further cost-benefit analytic studies need to be performed in order to determine the impact of such a proposed strategy as part of an integrated control programme.

## Supporting Information

Table S1 (0.04 MB DOC)

Checklist S1 CONSORT Checklist
(0.06 MB DOC)

Protocol S1 Trial Protocol
(1.03 MB DOC)
