# Supplementary material for: A Cluster-Randomised Intervention Trial against Schistosoma japonicum in the Peoples' Republic of China: Bovine and Human Transmission
Source: PLoS One. 2009 Jun 12;4(6):e5900. doi: 10.1371/journal.pone.0005900 (PMC2690852; doi:10.1371/journal.pone.0005900)
Supplement: Table S1 — (0.04 MB DOC) [file pone.0005900.s001.doc]

**Supplementary Table:** Baseline village pair characteristics

| Province | Hunan | | | | Jiangxi | | | |
| --- | --- | --- | --- | --- | --- | --- | --- | --- |
| Pair | Pair 1 | | Pair 2 | | Pair 3 | | Pair 4 | |
| Village Status | Control | **Intervention** | **Control** | **Intervention** | **Control** | **Intervention** | **Control** | **Intervention** |
| Administrative Village | **Yongxiang** | **Mengjiang** | **Jizhong** | **Yongfu** | **Fuqian** | **Xindong** | **Yu Feng** | **Cao Jia** |
| Location | Li Xian County,  Mengjiang Township | | Li Xian County,  Juiyuan Township | | Yugan County,  Kangshan Township | Yugan County,  Dongtang Township | Nanchang county, Jiangxiang township | Poyang county, Yingbaohu township |
| Area (km^2^) | 1.34 km^2^ | 1.23 km^2^ | 2.8 km^2^ | 2.4 km^2^ | 4 km^2^ | 5 km^2^ | 3 km^2^ | 4 km^2^ |
| Distance between villages within pairs (km) | 9 km | | 14 km | | 35 km | | 45 km | |
| Distance between village and water contact site (Lake) | 100–500 m | | | | 300 m | 100 m | 60 m | 100 m |
| Type of Marshland | Beach and Dyke Type | | | | Lake Type | | Lake Type | |
| Vegetation on Marshland | Grass and Reeds | | | | | | | |
| Registered Human Population | 1176 | 888 | 1581 | 918 | 1512 | 1649 | 1037 | 1262 |
| Actual Population Residing | 550 | 573 | 731 | 523 | 971 | 981 | 534 | 521 |
| Bovine  (Water Buffalo) Population | 68 | 61 | 97 | 112 | 257 | 250 | 108 | 59 |
| Goats present | Yes | Yes | Yes | Yes | No | No | No | No |
