# Supplementary material for: A Cluster-Randomised Intervention Trial against Schistosoma japonicum in the Peoples' Republic of China: Bovine and Human Transmission
Source: PLoS One. 2009 Jun 12;4(6):e5900. doi: 10.1371/journal.pone.0005900 (PMC2690852; doi:10.1371/journal.pone.0005900)
Supplement: Protocol S1 — Trial Protocol (1.03 MB DOC) [file pone.0005900.s003.doc]

# Protocol

# A Cluster-randomised Bovine Intervention Trial, against *S. japonicum* in the Peoples’ Republic of China

# NHMRC/Wellcome Trust

National Institute of Parasitic Diseases, Chinese Center for Disease Control and Prevention, Shanghai, China

Queensland Institute of Medical Research, Australia

School of Population Health, The University of Queensland, Australia

# Protocol Bovine Intervention Trial

PARTICIPATING SITE(S)

National Institute of Parasitic Diseases, China, CDC

Queensland Institute of Medical Research, Australia

School of Population Health, The University of Queensland, Australia

STUDY MANAGEMENT

All questions concerning this protocol should be sent via email to Darren Gray d.gray@sph.uq.edu.au

SCHEMA

It has been hypothesized that bovine, especially buffalo, infections are responsible for the persistence of human schistosomiasis transmission in the lake and marshland areas of China. A TMRC/NIH-supported pilot intervention study in the Poyang Lake region in the Jiangxi Province concluded in 2003 and the results are supportive of this hypothesis. The bovine intervention trial aims to: 1) Repeat the results obtained from the TMRC/NIH-supported pilot intervention study. However on a larger and more generalizable scale

; 2) Examine the efficacy of bovine chemotherapy on human infection and reinfection rates; 3) Assess bovine chemotherapy as a plausible schistosomiasis control method;

4) Integrate the intervention into the earlier TMRC developed mathematical model. The study will be conducted over a maximum of 4 years, with an interim analysis after 2 years within pairs of villages.

TITLE: A bovine intervention trial

COUNTRY: China

DURATION TO COMPLETION: 2006-2008

DESIGN: Cluster-randomized design with a longitudinal study of a sentinel cohort

SAMPLE SIZE: A sample size consisting of at least 300 residents per village will constitute the sentinel cohort; all bovines will be sampled in all pairs of villages at baseline and in the intervention villages during the trial.

POPULATION: The population will be from the pairs of selected administrative villages in Hunan and Jiangxi provinces in P.R. China

REGIMEN: The study is an intervention study. It will entail an initial treatment with Praziquantel of humans in all villages and bovines within the intervention villages only. Humans will be mass treated annually just after the transmission season and Bovines will be mass treated twice annually, just before and after transmission season. Prior to the annual mass treatment, identification of infected persons in the selected cohort, via standard stool examination (Kato-Katz method) will be undertaken. A name list of positives will be provided to the local control program and will be treated by local control teams according to standard protocols established within the schistosomiasis control program in China. This is necessary for ethical reasons.

**Contents**

**Page**

1. **Introduction 4**
   1. Background 4
   2. Rationale 6
   3. Study Design 7
2. **Study Objectives 7**
3. **Study Procedure 8**
   1. Modus Operandi 8
   2. Study Timetable 13
4. **Selection of Subjects 13**
   1. Village Selection 13
   2. Cohort Selection 15
5. **Clinical Considerations 16**
   1. Laboratory Evaluation 16
   2. Treatment Regime 17
   3. Adverse Reactions 18
6. **Data Collection 18**
   1. Records to be Kept 18
   2. Data Management 19
7. **Statistical Considerations 19**
   1. Outcome Measures 19
   2. Outcome Measure Formulae 19
   3. Statistical Significance, Generalizability and Power 21
   4. Analysis Plan 22
8. **Ethics 22**
   1. Humans 22
   2. Bovines 22
9. **Biohazard Containment 22**
10. **References 23**

APPENDIX 24

# Introduction

**1.1 Background**

600 million Chinese have parasitic infections. One of the most important, from the public health and clinical perspective, is schistosomiasis (Hotez et al., 1997). Schistosomiasis japonica is a serious disease, typified by long-term chronicity, and a major disease risk for more than 40 million people living in the tropical and sub-tropical zones of China (Chen and Feng, 1999). The disease disabled and killed millions of Chinese peasants before systematic control programs began in the 1950s (Chen, 1999). Infection remains a major public health concern despite the 45 years of extraordinary historic emphasis and intensive efforts at controlling this disease. Even today, it is estimated that over 1 million people and several hundred thousand livestock, including 100,250 bovines, are infected (Ross et al., 2001). The major endemic foci are in the marsh and lake regions (Dongting and Poyang Lakes; **Figure 1**) of Southern China, which cover a vast area of five provinces, Jiangsu, Anhui, Hubei, Jiangxi and Hunan. Since 1985, the rural Chinese economy has been boosted (resulting in an improved standard of living) but the prevalence of *S. japonicum* and its associated morbidity have also risen in focus areas (Yuan, 1992). Unlike the other schistosome species known to infect man, the oriental schistosome, *Schistosoma japonicum*, is zoonotic, with a range of mammalian reservoirs that complicates control efforts. Schistosome infection debilitates domestic livestock that are used for food and as work animals in Southern China. This adds to the economic burden and suffering of communities in the *S. japonicum*-endemic areas.


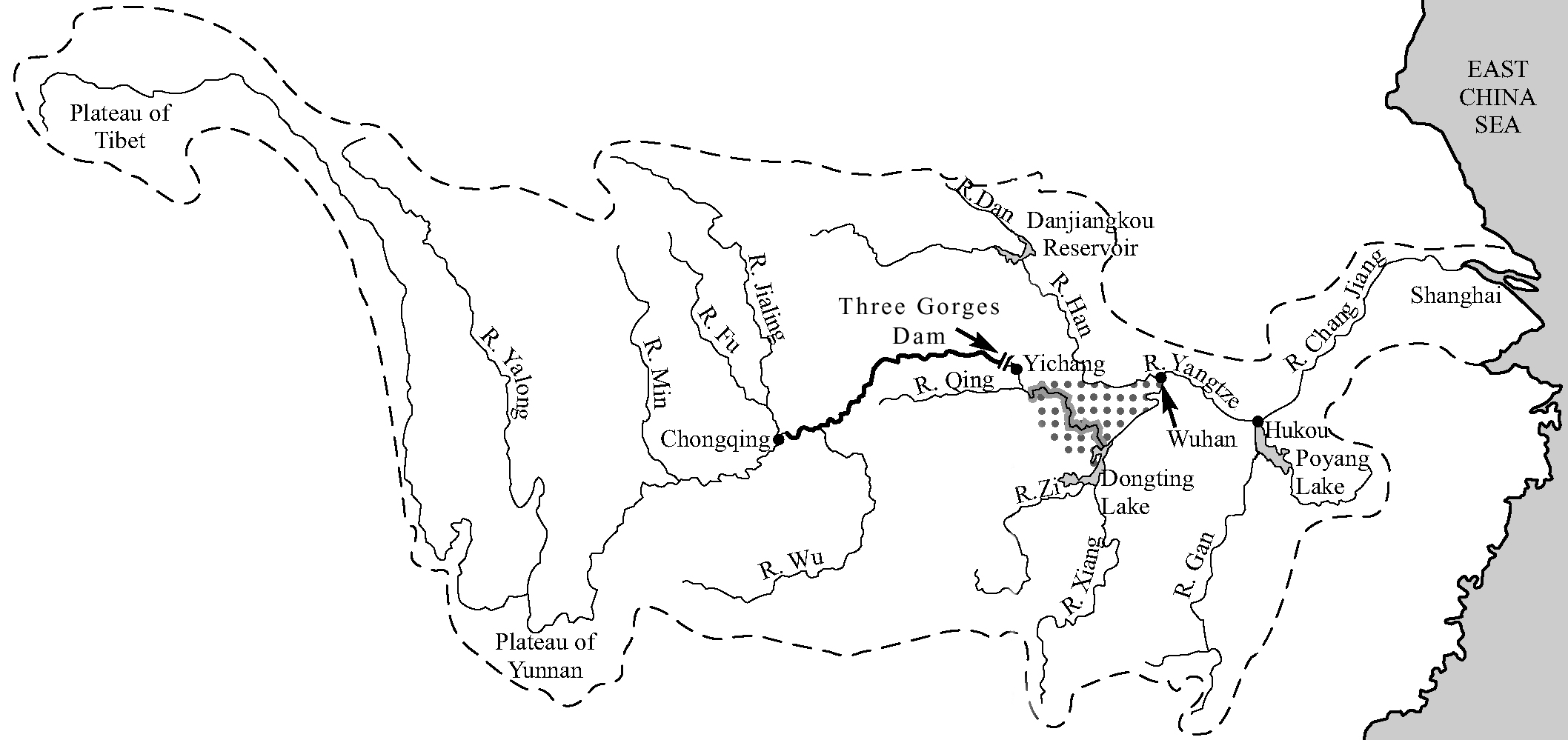


**Figure 1. Map of Southern China showing the position of the Three Gorges Dam and Poyang and Dongting Lakes on the River Yangtze**.

Clinical features of schistosomiasis japonica are severe, ranging from fever, headache and lethargy, to serious fibro-obstructive pathology leading to portal hypertension, ascites and hepatosplenomegaly, which can cause premature death (Ross et al., 2002). As well, infected children are stunted and have cognitive defects impairing memory and learning ability. The onset of advanced schistosomiasis poses a particular public health and socio-economic problem. There are over 30,000 cases of advanced disease with 8,000 new cases reported annually. The onset of advanced disease promotes the most serious clinical manifestations and it is difficult to provide adequate treatment for affected patients, many of whom die resulting in severe consequences both for the patient’s family and the community as a whole. Current control is based on community chemotherapy with a single dose of praziquantel. Vaccines (for use in bovine and humans) in combination with other control strategies, including the use of new drugs, are needed to make elimination of the disease possible (Todd & Colley, 2002). New approaches, advocated by the Chinese Government and other organizations including WHO, are required to supplement current control strategies that are difficult and costly (Guyatt, 1998). We recently brought together the results of control technology advances, including the success of recent World Bank inputs, to mathematically model prospects for the future control of schistosomiasis in China (Williams et al., 2002). The model shows that an integrated approach to control can successfully lead to eradication of infection.

China is in the midst of major environmental and social change and a further important issue concerns construction of the giant Three Gorges Dam **(Figure 1)** across the Yangtze (Chang Jiang) River, whose drainage provides the great corridor of parasitic disease in China. Due to be filled by 2009, the dam, which was recently closed, and the 600 km long reservoir it will create, will have a global environmental impact on the transmission and geographical distribution of schistosomiasis both above and below the dam (Zheng et al., 2002). The Ministry of Health in China is currently investigating the increased risk for schistosomiasis associated with the dam. No absolute schistosomiasis data exist to indicate the probable outcomes but there is no doubt the dam will change the Yangtze basin ecology significantly. A second wave of problems has been caused by a plague of recent floods that have devastated numerous regions of the Yangtze River drainage. The recent flood problems are associated with increasing deforestation in the mountains coupled with reclaiming of lakes and marshy lowlands in the Lake Districts in the lower Yangtze drainage. This will increase snail habitats and, consequently, the risk of schistosome transmission. Over the coming years the numbers of oncomelanid-snail breeding areas will be increased substantially and schistosomiasis transmission will intensify. As a result we predict a marked elevation in infection rates and associated morbidity that will impact significantly on control.

## 1.2 Rationale

Bovines (cattle and water buffalo) are the major reservoir of *S. japonicum* within the marsh and lake regions of China. A TMRC-supported pilot intervention study (1998-2003) around the Poyang Lake, Jiangxi Province aimed at providing proof-of principle for the hypothesis that bovine, especially buffalo, infections are responsible for the persistence of human schistosomiasis transmission in the marshlands and lake areas of China. The work has major implications for future integrated schistosomiasis control including the use of chemotherapy for bovines and it underpins the rationale for development and implementation of a veterinary-based vaccine for use in reservoir hosts, particularly buffaloes. This pilot study (1998-2003) was comparing human infection incidence in an intervention village (Jishan) (in which bovines were treated) and a control village (Hexi) (no bovine treatment) (Guo et al., 2001; Davis et al., 2002). We found that the incidences of infection in residents and bovines were reduced significantly in the intervention village. We used the predicted human infection incidence data in our mathematical model (Williams et al, 2002) based on data collected in Jishan village to compare predicted prevented infections associated with bovine treatment (**Figure 2**).


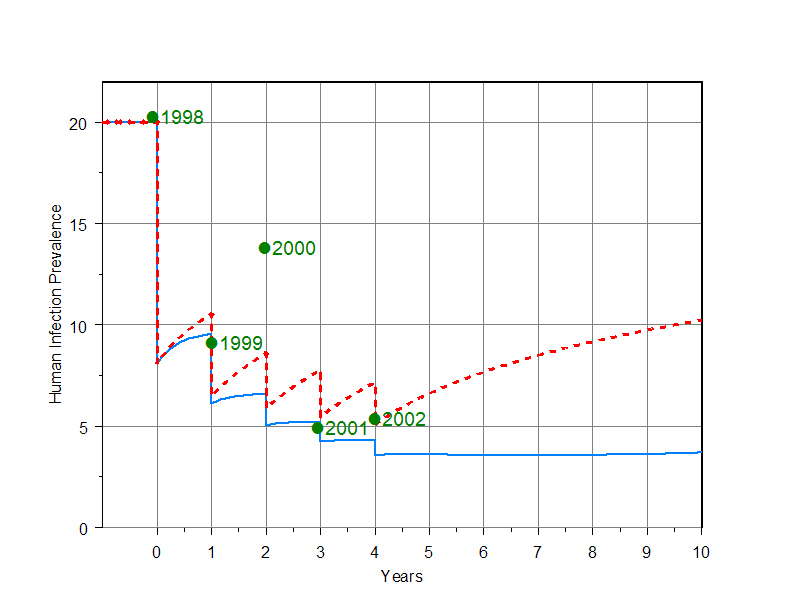
**Figure 2.** **Predicted human infection prevalence from the mathematical model, based on data collected in Jishan village, for human treatment only (broken line) and human and bovine treatment combined (solid line). Solid dots show the prevalences actually observed in Jishan. The gap between the two curves represents predicted prevented infections associated with bovine treatment.**

***Aim:*** The pilot study provides proof of principle but we wish now to undertake a more stringent bovine intervention trial (2004-2008) using a cluster-randomized design, with increased power and general application.

### 1.3 Study Design

**Cluster Randomized Design:** We will select pairs of villages from identified communities (human prevalence 10-15%; bovine prevalence 10-15%) in Jiangxi province and Hunan province, with pairs being matched as closely as possible on factors related to transmission: i.e. infection levels, transmission ecology (e.g.: water height, flooding patterns), and force of infection (e.g.: water contact patterns, buffalo: human numbers, sanitation practices, herding practices). The trial will aim to select the villages that provide closest matching in pairs. Within each pair, one village will be randomly chosen as intervention (bovines treated twice annually) and the other will be a control. Annual mass treatment of residents will be carried out in both villages. The end-point for the trial will be human infection rates. The efficacy of bovine treatment will be measured by the accumulated comparison of human infection rates across intervention and control villages, and will be reflected in the predicted percentage of human infection-years prevented by the intervention. Human infection rates will be assessed at the end of each year for 4 years.

### 2.0 Study Objectives

This study aims to achieve the following objectives.

1. Repeat the results obtained from the TMRC-supported pilot intervention study, regarding the hypothesis that “Bovine, especially buffalo, infections are responsible for the persistence of human schistosomiasis transmission in the marshlands and lake areas of China.” However on a larger and more generalizable scale.
2. Examine the efficacy of bovine chemotherapy on human infection and reinfection rates and how this gives insight into the use of a potential bovine vaccine.
3. Assess bovine chemotherapy as a plausible schistosomiasis control method, particularly in combination with human treatment.
4. Integrate the empirical data collected from the study into the previously TMRC-developed mathematical model.
5. **Study Procedure**

**3.1 Study procedure**

##### Pre-Trial

A research team in each province will be established in order to carry out the fieldwork and the laboratory testing in their respective research institutes.

A sampling survey in Jiangxi Province will be done by early September 2004. The data from this survey will be distributed to the project investigators for the selection of the study villages.

Approximately 40-50 human faecal samples and 30 bovine faecal samples per village will be collected. Additionally, during the sampling survey, faecal examinations for sheep and goats (40-50 each species, hatching method) will be carried out to estimate their prevalence, as a variable for modelling.

Maps of the study sites will be prepared in order to show their location.

After the Jiangxi sampling survey, the study villages will be selected and matched according to the village selection criteria, by the project investigators. Within each pair, one village will be randomly selected as the intervention village thus leaving the other of the pair as the control.

A current list (census) of each village population by household will be constructed prior to baseline

**Stage 1:** October 2004

##### Baseline Survey

##### Humans

This will include a questionnaire-based interview whereby demographic information (including name, sex, date of birth, length of time resident in the village), water contact information, and a brief medical history (schistosomiasis) will be obtained. Each person will be assigned a personal identification number (PID) and entered into a case report form (CRF). Their PID will be kept for the duration of the study.

All village residents will receive 2 plastic containers for fecal samples. Containers will be labeled with PID, and the date of distribution. Participants will be instructed to deposit samples from their own faeces, separated by at most three days, into each container and to return the container immediately to one of several collection points, where the sample will be stored at 4 °C. Fecal samples returned later than 48 hours after defecation will not be accepted and new containers will be issued. Slide preparation (three slides per stool specimen) will occur 24 hours after receipt of stool sample using the Kato-Katz thick smear technique (Katz et al 1972). These samples will provide the baseline prevalence and intensity of infection within each village.

Anyone found to be egg positive would be treated for ethical reasons.

##### Bovines

A baseline survey will also be carried out on the bovines within the villages. A stool sample will be obtained from all bovines and tested, using the traditional Chinese miracidial hatching test in order to determine the baseline prevalence and intensity of infection within each village. Sheep and goats will also be examined at baseline

Permission will be obtained from the bovine owners to allow us to treat their bovines. Note this will only happen in the intervention villages.

Note: All Bovines will be examined and treated in the intervention villages; they will only be examined in the control villages.

##### Snails

Want to measure the prevalence of infection in snails and the density of infected snails per unit area. To do this we need to conduct random quadrat sampling (1000 frames 20-50m per frame) (Chinese method) of the marshland areas relevant to each village. Use GPS machine, to identify the area that was surveyed.

This will need to be done at baseline, early October.

Note: Snail surveys will also be done and in March/April each year.

##### Sentinel cohort selection

Within each village a sentinel cohort will be selected and followed up on for a maximum of 4 years. This cohort will be selected by the following procedure:

The cohort will be selected for the study based on the inclusion criteria. The project’s principal investigators will perform the selection. The sentinel cohort will comprise at least 300 residents per village.

The research team will then visit the households of those selected in order to recruit them for the study and to obtain informed consent from individual participating adults and assent for minors. The subject (or parent or legal guardian if the subject is less than eighteen years of age) will be asked to read and sign an informed consent approved by the IRB. Their personal identification number (PID) from the baseline survey will be entered on their consent form prior to its signing. It must be noted that at the start of the study there must be a minimum of 300 residents in each cohort.

**Stage 2:** November 2004

##### Mass Treatment

All village residents will be treated with Pzq as per the treatment regime outlined below.

Stool samples will be taken from sentinel cohort members 1 month after the treatment to determine the efficacy of treatment. Those found to be egg positive would be retreated as per the regime. This process will be repeated until the subject is cured of infection.

All bovines within the intervention villages will be treated and cured with Pzq as per the treatment regime outlined below. Stool samples will be taken 1 month after the treatment to determine the efficacy of treatment. Those found to be egg positive would be retreated as per the regime. This process will be repeated until the animal is cured of infection.

**Stage 3:** March/April 2005 to May 2005

##### Snail survey

##### March/April 2005

Want to measure the prevalence of infection in snails and the density of infected snails per unit area. To do this we need to conduct random quadrat sampling (1000 frames) (20m^2^ or 50m^2^ depending on area)(Chinese method) of the marshland areas relevant to each village. Use GPS machine to provide a snail survey map

##### Mass Treatment

May 2005

All bovines within the intervention villages will be treated and cured with Pzq as per the treatment regime outlined below. Stool samples will be taken 1 month after the treatment to determine the efficacy of treatment. Those found to be egg positive would be retreated as per the regime. This process will be repeated until the animal is cured of infection.

**Stage 4:** October-November 2005

##### Outcome Measures

Humans:

Sentinel cohort members will receive 2 plastic containers for fecal samples. Containers will be labeled with PID, and the date of distribution. Participants will be instructed to deposit samples from their own faeces, separated by at most three days, into each container and to return the container immediately to one of several collection points, where the sample will be stored at 4 °C. Fecal samples returned later than 48 hours after defecation will not be accepted and new containers will be issued. Slide preparation (three slides per stool specimen) will occur 24 hours after receipt of stool sample using the Kato-Katz thick smear technique (Katz et al 1972). These samples will provide the outcome measures for each village 1 year after baseline. Water contact data will also be collected for that month via the completion of a questionnaire

Cohort members found to be egg positive will be treated for ethical reasons.

Water contact questionnaire survey

The questionnaire will be conducted once a year after transmission season (October-November) by trained team members, no more than 3 team members will be responsible for the survey for one village during the entire project period. The content of the water contact survey is listed in the questionnaire form. Each item must be answered with no blank.

Bovines:

A stool sample from every bovine will be collected and tested using the traditional Chinese MHT, These samples will provide the outcome measures for each village 1 year after baseline.

**Stage 5:** November 2005

##### Mass Treatment

All village residents will be treated with Pzq as per the treatment regime outlined below.

Stool samples will be taken from sentinel cohort members 1 month after the treatment to determine the efficacy of treatment. Those found to be egg positive would be retreated as per the regime. This process will be repeated until the subject is cured of infection.

All bovines within the intervention villages will be treated and cured with Pzq as per the treatment regime outlined below. Stool samples will be taken 1 month after the treatment to determine the efficacy of treatment. Those found to be egg positive would be retreated as per the regime. This process will be repeated until the animal is cured of infection.

**Stage 6:** March/April to May 2006

##### Snail survey

##### March/April 2006

Want to measure the prevalence of infection in snails and the density of infected snails per unit area. To do this we need to conduct random quadrat sampling (1000 frames) (20m^2^ or 50m^2^ depending on area)(Chinese method) of the marshland areas relevant to each village. Use GPS machine to provide a snail survey map

##### Mass Treatment

May 2006

All bovines within the intervention villages will be treated and cured with Pzq as per the treatment regime outlined below. Stool samples will be taken 1 month after the treatment to determine the efficacy of treatment. Those found to be egg positive would be retreated as per the regime. This process will be repeated until the animal is cured of infection.

**Stage 7:** October-November 2006

##### Outcome Measures

Humans:

Sentinel cohort members will receive 2 plastic containers for fecal samples. Containers will be labeled with PID, and the date of distribution. Participants will be instructed to deposit samples from their own faeces, separated by at most three days, into each container and to return the container immediately to one of several collection points, where the sample will be stored at 4 °C. Fecal samples returned later than 48 hours after defecation will not be accepted and new containers will be issued. Slide preparation (three slides per stool specimen) will occur 24 hours after receipt of stool sample using the Kato-Katz thick smear technique (Katz et al 1972). These samples will provide the outcome measures for each village 2 years after baseline. Water contact data will also be collected for that month via the completion of a questionnaire

Cohort members found to be egg positive will be treated for ethical reasons.

Water contact questionnaire survey

The questionnaire will be conducted once a year after transmission season (October-November) by trained team members, no more than 3 team members will be responsible for the survey for one village during the entire project period. The content of the water contact survey is listed in the questionnaire form. Each item must be answered with no blank.

Bovines:

A stool sample from every bovine will be collected and tested using the traditional Chinese MHT, These samples will provide the outcome measures for each village 2 years after baseline.

**Stage 8:** November 2006

##### Interim Analysis

An interim analysis of the results will be performed using Bayesian methods to determine if early termination of the project is possible.

If early termination is possible, a final analysis will be performed.

However, if termination is not possible the project will move on to stage 9.

**Stage 9:** November 2006 – October-November 2008

Stages 2-7 will be repeated.

**Stage 10:** November 2008

##### Final Analysis

Final results analysis

**3.2 Study Timetable**

| **Stage 1**  **(October 2004)** | **Stage 4**  **(October 2005)** | **Stage 7**  **(October 2006)** | **Stage 9**  **(October 2007)** | **Stage 9**  **(October 2008)** |
| --- | --- | --- | --- | --- |
| **Stage 2**  **(November 2004)** | **Stage 5**  **(November 2005)** | **Stage 8**  **Or**  **Stage 9**  **(November 2006)** | **Stage 9**  **(November 2007)** | **Stage 10**  **(November 2008)** |
| **Stage 3**  **(March-May 2005)** | **Stage 6**  **(March-May 2006)** | **Stage 9**  **(March-May 2007)** | **Stage 9**  **(March-May 2008)** |  |

**4.0 Selection of Subjects**

**4.1 Village selection**

- The villages (administrative and/or natural) identified, from which the study villages will be selected from need to exhibit similar characteristics; in particular:
  - The prevalence and intensity of infection at baseline within humans and bovines (particularly Buffalo). Human prevalence 10-15% and bovine prevalence 10-15%.
  - Similar transmission factors (i.e. transmission ecology and force of infection)
  - Sufficient populations of humans and bovines in order to obtain a successful and conclusive study. Approximate numbers of humans and bovines would be 1000 and 100 respectively.
  - These identified villages must be separate from any other studies (e.g. the TMRC 2 study)
  - Ideally the identified villages will be from the lake and marshland districts of the provinces Hunan and Jiangxi.
  - Bovines are separate from other villages
  - The villages are sufficiently isolated from one another
  - Village will not be relocating in the next 4 years.
- From the identified villages pairs will be selected from Hunan province and from Jiangxi province.
- Each pair will be matched as closely as possible on the following characteristics.

###### Essential

- - The matched pairs are from the same province (i.e. 2 matched pairs from Hunan, 2 matched pairs from Jiangxi).
  - The human to bovine ratio is similar within the pair.
  - The prevalence and intensity of infection at baseline within humans and bovines (particularly Buffalo) are similar.
  - The ecology of the villages must be similar (e.g. climate, water height, flooding patterns (rainfall), vicinity to lakes and/or marshlands).
  - Grazing of the bovines is within their village.
  - The villages are sufficiently isolated from one another.
  - Water contact patterns are similar.
  - No boat harbors within the village, where fishing boats frequently visit leading to the contamination of marshland by defecation of infected fishermen.

Desirable

- - Village layout and geographical size are similar.
  - Occupations of residents are similar.
  - Sanitation practices are similar.
  - Herding practices are similar.

### 4.2 Cohort Selection

# Cohort Inclusion Criteria

1. Resident of the administrative village and/or natural village selected for study.
2. Has been a resident of the village for >12 months.
3. 5-60 years of age.
4. Will not be migrating in the next 4 years.
5. Those who continuously reside in the study area over the study period^*^.
6. The resident has given informed consent.
7. Minors have the informed consent of their parent/guardian.

* Residents who are in the study area only weekends or once a month (such as students) are assumed not to have the same kind or an equal amount of exposure as individuals continuously resident in the study area. Inclusion of such individuals would contribute to either an overestimation or underestimation of prevalence.

Out-migration (dates and reasons) will be recorded for cohort members. Owing to the close familial relationships within the village, reporting of out-migrants will rely on family members or neighbours.

In addition, in-migrants to the area, intending to become longer-term residents will be documented, but not included in the cohort. Thus, the study will have documentation of the village population, within which the cohort is embedded.

NOTE: The sentinel cohort in each village must have at least 300 members

1. **Clinical Considerations**

**5.1 Laboratory evaluation**

##### Humans

Human fecal analysis will be performed using the Kato-Katz thick smear technique (Katz et al 1972.), 2 stools will be collected per person and 3 slides will be prepared per stool. Each smear will be read blind from the previous and each stool will be read blind from the previous.

Fecal sample containers will be labeled with the PID, and the date of distribution. Participants will be instructed to deposit samples from their own feces, separated by at most three days, into each container and to return the container immediately to one of several collection points, where the sample will be stored at 4 °C. Fecal samples returned later than 48 hours after defecation will not be accepted and new containers will be issued. The slide preparation will occur 24 hours after receipt of stool sample.

The term “egg positive” for *S. japonicum* will be defined as a single egg found in any one of the 6 fecal thick smear slides. Cohort members found to be egg positive will be treated for ethical reasons.

Any cohort member who receives treatment whether it is as part of the annual mass treatment or as a direct result of being egg positive will have to provide stool samples as explained above 1 month after treatment to test the efficacy of that treatment.

Results of the fecal analysis will be recorded on the human stool examination forms shown in the appendix.

##### Bovines

Bovine fecal analysis will be performed using the traditional Chinese miracidial hatching test and their sedimentation method. The miracidial hatching test will determine whether or not the bovine is positive or negative for *S. japonicum* and their sedimentation method will determine the intensity of infection.

One stool will be collected per animal with 3 hatches (50 g faeces/hatching), which are to be read blind from each other.

The stool is to be collected from the animals’ stable or directly from its rectum.

The research team will collect the stool for examination into containers labeled with the date of collection and the type of animal with its PID.

Any bovine which receives treatment whether it be as part of the twice annual mass treatment or as a direct result of being egg positive will have to have a stool sample collected 1 month after treatment to test the efficacy of that treatment.

Results of the fecal analysis will be recorded on the bovine stool examination forms shown in the appendix.

**5.2 Treatment Regime**

Humans

All village residents will be treated with an annual single dose of praziquantel (Pzq) 40mg/kg. Participants will be observed for 15 minutes after taking the medication.

Stool samples will be taken from sentinel cohort members 1 month after the treatment to determine the efficacy of treatment. Those found to be egg positive would be retreated with a single dose of Pzq 40 mg/kg. This process will be repeated until the subject is cured of infection.

Note: Previous studies in Poyang and Dongting Lakes show 85% efficacy on a single praziquantel dose, with 100% efficacy following re-examination and re-treatment as described above (Li et al, 1999).

Interloping fisherman and boatmen are to be treated twice annually if applicable and possible with Pzq 40mg/kg.

## Bovines

All bovines in the intervention villages will be treated twice annually with a single dose of praziquantel (Pzq) until cured. Buffaloes are to be treated with Pzq 25mg/kg and cattle are to be treated with Pzq 30mg/kg.

Stool samples will be taken 1 month after the treatment to determine the efficacy of treatment. Those found to be egg positive would be retreated with a single dose of Pzq (buffaloes 25mg/kg, cattle 30mg/kg). This process will be repeated until the animal is cured of infection.

All new bovines to the intervention village will be tested and treated as per the above regime and this will be recorded. Additionally newborn bovines will be recorded and treated 3 months after birth.

Neighbouring bovines found to be on the study sites’ marshland will be regarded as if they are part of the study group and hence will be treated accordingly. They will not be included in the calculation of prevalence.

Note: 100% treatment coverage must be achieved for the bovines.

Other mammals such as sheep and goats will also be treated twice annually in both control and intervention villages by single dose of 20 mg/kg. Moreover, if dogs and pigs are roaming on the marshland they too will be treated with Pzq 20mg/kg in both control and intervention villages.

# Adverse reactions

##### Humans

Treatment by praziquantel has been a routine, accepted, well-tolerated and widely used measure to control schistosome infection in all endemic areas of China. Treatment with praziquantel is safe, with very limited side effects. In the very unlikely event of minor side effects such as headache, medical treatment will be offered free of charge immediately. Participants in the project are at no greater risk than when they join the local schistosomiasis control program or see doctors in local clinics.

# Bovines

In the event of a bovine death because of the Pzq, the owner will be compensated for the loss.

**6.0 Data Collection**

- 1. **Records to be kept**

##### Humans

Case Report Forms (CRF) CRFs will be kept for each subject, for each data collection, i.e.

human survey and human fecal examination. Subjects will be identified by their administrative

village, natural village, house identification, and an individual identification within the house. In

addition they will be assigned a unique serial number which will be constant over time, and

enable longitudinal analysis. CRFs will be completed according to standard operation procedures

(SOPs). That is, all data on the CRF will be legibly recorded in black ink, corrections may only

be made by striking through an incorrect entry with a single line and entering the correct

information adjacent to it. The correction will be initialed and dated by the investigator or a

designated, qualified individual.

A migration CRF will be used to record exit from the study, the date of exit, and the reason.

In-migrants will not be recruited to the study, however their entry to the village will be recorded along with the date of entry, and the reason. Furthermore, these in-migrants will be tested and treated on entry.

Note: Only those who enter the study site during the transmission season and stay longer than two weeks will be examined and treated. If from an endemic area or are having water contact, need to be examined and treated. If living in the village, need to be examined and treated. All will be recorded.

##### Bovines

Case report forms will also be kept for the bovines. Their administrative village, natural village, house identification, host identification, animal type, sex and age will identify the bovines. In addition, they will be assigned a unique serial number, which will be constant over time, thus

enabling longitudinal analysis. A stool examination form will record the results of the fecal testing; a migration section will be included on the stool examination form and it will be used to record exit from the study, the date of exit, and the reason. In addition, it will be used to record entry into the study, the date of entry, and the reason. The recording of the bovine data will follow the same SOP as that of the recording of the human data.

- 1. **Data Management**

A database will be designed using Microsoft Access in order to manage the data. Each research team will double enter the data at their respective research institute. The CRFs used to enter the data from will be stored in a secure, fireproof place in case they need to be reused and to protect the identities of participants. It is important that the same procedure is used to enter the data and the same database is used across all research teams and their research institutes in order to standardize this process.

**Statistical Considerations**

**7.1 Outcome Measures**

The outcome measures for this study include the prevalences, infection and reinfection rates, intensities of infection and incidences. The end-point for the trial will be human infection rates. The efficacy of buffalo treatment will be measured by the accumulated comparison of human infection rates across intervention and control villages, and will be reflected in the predicted percentage of human infection-years prevented by the intervention. Human infection rates will be assessed at the end of each year for 4 years.

**7.2 Outcome Measure Formulae**

Prevalence = No. of egg positive cases

No. persons at risk × 100

For the prevalence per village, the number of persons at risk would equal the village population.

Incidence of infection : Since all positive cases are treated (and confirmed negative) at the end of

each year, the incidence rate of infection for a year is the number of infected persons in the cohort

found at the end of each year (having been present during the year), divided by the number of

persons in the cohort present during the period.

Year K Incidence density = No. of infections at end of year K _____ × 100

(K=2 to 5) No. of persons in cohort at end of years K and K-1

To take account of drop-outs, where additional information is available on the timing, we will be

able to refine this estimate by using a ‘person-time denominator’, which modifies his or her

exposure time by drop-out time: person-time = date of drop-out – date of start of period:

Incidence of newly-infected: This measure examines the rate of infection in those not infected at

the end of the previous period (year). It is the number of infected persons in the cohort found at

the end of each year (having been present during the year), divided by the number of persons in

the cohort present during the period, and negative at the end of the previous year.

Year K Newly-infected = No. of infections at end of year K__________ × 100

(K=2 to 5) No. of persons in cohort at end of

years K and K-1, and negative at end of year K-1

Rate of reinfection: This measure examines the rate of infection in those infected at the end of

the previous period (year). It is the number of infected persons in the cohort found at the end of

each year (having been present during the year), divided by the number of persons in the cohort

present during the period, and positive at the end of the previous year.

Year K Re-infected = No. of infections at end of year K × 100

(K=2 to 5) No. of persons in cohort at end of years K and K-1,

and positive at end of year K-1

Intensity of reinfection: The intensity of infection for new cases and the intensity of reinfection

will be determined by the number of eggs per gram of feces, which is measured using the Kato-Katz thick smear technique.

**7.3 Statistical significance, Generalizability and Power**

In order for this study to yield results that are statistically significant and are able to be applied to a population (i.e. generalizable), there needs to be adequate power and a sample size providing an accurate representation of the affected community. It has been determined that for generalizability and statistical significance, a minimum of 4 village pairs is required, with a sample size of 300 per village, with a village population of approximately 1000.

The following table shows the power calculations based on the above minimum considerations. The

table also shows these powers with varying degrees of the bovine effect on the village and the

percentage of the bovines that are actually treated.

Assumptions

- Design effect is 1.5, in other words the actual sample size is reduced by a factor of 1.5 to give an effective sample size. This reduction is due to the extra similarity within clusters that means the observations cannot be treated as independent.
- Assume an attrition rate between years 1 and 2 of 20%.
- Assume that years 1 and 2 are independent for the cumulative power.
- Target (net) sample size is the number obtained in each village for testing (after excluding non-compliers).
- Use a significance level of 1% year 1 and 5% year 2.
- This study works on treating the whole village.
- Assume a village size of 1000.

# Table of sample size (no new villages)

| Bovine effect size &  % Bovines treated | # Paired villages | Target sample size per village | Power %  (Year 1) | Power %  (Year 2) | Cumulative power  (Yrs 1 & 2) |
| --- | --- | --- | --- | --- | --- |
| 60% & 70% | 4 | 300 | 26.7 | 80.3 | 89.5 |
| 60% & 80% | 4 | 300 | 38.1 | 88.7 | 95.7 |
| 60% & 90% | 4 | 300 | 51.3 | 94 | 98.5 |
|  |  |  |  |  |  |
| 70% & 70% | 4 | 300 | 38.2 | 90.1 | 96.2 |
| 70% & 80% | 4 | 300 | 53.3 | 95.5 | 98.9 |
| 70% & 90% | 4 | 300 | 68.6 | 98 | 99.8 |
|  |  |  |  |  |  |
| 80% & 70% | 4 | 300 | 50.9 | 95.7 | 98.9 |
| 80% & 80% | 4 | 300 | 68.0 | 98.4 | 99.8 |
| 80% & 90 % | 4 | 300 | 82.7 | 99.4 | 100 |

Target sample size per village corresponds to the number of villagers sampled (not necessarily all those exposed). Powers are based on adjusting the sample size per village for attrition and a design effect.

- 1. **Analysis Plan**

Initial descriptive statistics will be performed to examine an overall trend in the data. This will be followed by the calculation of the outcome measures. These outcome measures will then be compared across control and intervention villages in order to determine the efficacy of the intervention. The empirical data gathered in this study will be put into the previously developed mathematical model (Williams *et al* 2002) in order to validate its predictions. Microsoft Access will be used for Data Management and SAS will be used for Data Analysis.

1. **Ethics**

**8.1 Humans**

The project has received IRB review and approval from the Chinese Institute of Parasitic

Diseases and the Queensland Institute of Medical Research. Written informed consent will be obtained from each eligible adult participant and assent for children will obtained through their primary caretakers, as described above (see Appendix). Identifying information, such as names will be kept confidential, and all CRF’s will be kept secure at all times.

Those found to be infected at any time by *S. japonicum* will be referred to the local control program and will be treated by local control teams according to standard protocols established within the schistosomiasis control program in China.

- 1. **Bovines**

The project has received IRB review and approval from the Chinese Institute of Parasitic

Diseases and the Queensland Institute of Medical Research. Informed consent will be obtained from the owner to use their bovines in the study. In the event of a bovine death because of the Pzq treatment, the owner will be compensated for the loss.

1. **Biohazard Containment**

No blood samples will be used in this project. The only biological samples will be human and

animal fecal specimens which will be handled according to appropriate protocols within the

schistosomiasis control program in China.

**10.0 References**

Chen MG. Progress in schistosomiasis control in China. *Chinese Medical Journal* 1999;112: 930-933.

Chen MG, Feng Z. Schistosomiasis control in China. *Parasitology International* 1999; 48:11-19.

Hotez, PJ, Zheng, F, Long-qi, X, Ming-gang, C, Shu-hua, X, Shu-xian, L, Blair, D, McManus,

DP and Davis, GM. Emerging and reemerging helminthiases and the public health of China.

*Emerg Infect Dis* 1997; 3: 303-10.

Ross, AGP, Bartley, PB, Sleigh, AC, Olds, GR, Li, YS, Williams, GM and McManus, DP.

Schistosomiasis. *New England Journal of Medicine* 2002; 1212-1220.

Ross, AGP, Sleigh, AC, Li, Y, Davis, GM, Williams, GM, Jiang, Z, Feng, Z and McManus, DP.

Schistosomiasis in the People's Republic of China: prospects and challenges for the 21st

Century. *Clinical Microbiology Reviews* 2001; 270-295.

Williams, GM, Sleigh, AC, Li, YS, Feng, Z, Davis, GM, Chen, H, Ross, AGP, Bergquist, R and

McManus, DP. Mathematical modelling of *Schistosoma japonica*: comparison of control

strategies in the People’s Republic of China. *Acta Tropica* 2002; 2: 253-262.

Yuan HC Epidemiological features and control of schistosomiasis japonica in China. *Memorias Instituto Oswaldo Cruz* 87:241.

Zheng J, Gu XG, Xu YL, Ge JH, Yang XX, He CH, Tang C, Cai KP, Jiang QW, Liang YS, Wang TP, Xu XJ, Zhong, JH, Yuan HC, Zhou XN. Relationship between the transmission of schistosomiasis japonica and the construction of the Three Gorges Reservoir. *Acta Tropica* 2002; 82:147-56.

Katz N, Chaves A, Pellegrino J (1972): A simple device for quantitative stool thick-smear

technique in Schistosomiasis mansoni. Rev Inst Med Trop Sao Paulo. 14(6): 397-400.

Li, YS., AC. Sleigh., AGP. Ross., Y. Li., GM Williams., SJ. Forsyth., M. Tanner and DP.

McManus (1999). A two-year epidemiological survey in China provides epidemiological

evidence for human resistance to re-infection by *Schistosoma japonicm.* Annals of Tropical

Medicine & Parasitology 93, 629-642.

### APPENDIX I

**FORM 1**

**Human Survey (Baseline)**

## ID Information

Name___________________________________________________________

Province Administration village Natural village House Individual

|__| |__| |__|__| |__|__|__| |__|__|

Respondent for interview: |__| (1) Self; (2) Parent; (3) Child;

(4) Brother/sister; (5) Spouse; (6) Other

# Demographic Information

Sex |__| M/F Birth date |__|__|__|__|/|__|__|/|__|__| yyyy/mm/dd

Education |__| (1) Illiterate; (2) Semi-literate; (3) Primary school

(4) Junior School; (5) Senior School; (6) College

Years of education: |__|__|

Occupation: |__| (1) Farmer; (2) Fisherman; (3) Farmer + Fisherman; (4) Businessman; (5) Teacher; (6) Student; (7) Pre-School; (8) Other

# Schistosomiasis history

Have you ever had

Schistosomiasis? No N Yes Y Don’t know D

Advanced schistosomiasis? No N Yes Y Don’t know D

Acute schistosomiasis? No N Yes Y Don’t know D

Number of previous treatment for the schistosomiasis? |__||__| times

Have you had schistosomiasis in 2 years? No N Yes Y Don’t know D

In the last 2 weeks have you had:

Diarrhea? 0 No; 1 Often; 2 A few times; 3 Don’t know

Weakness? 0 No; 1 Often; 2 A few times; 3 Don’t know

Fever? 0 No; 1 Often; 2 A few times; 3 Don’t know

**Other Medical Information**

Hepatitis B: Have you ever had Acute hepatitis? Y/ N/ Unsure

Have you ever had Chronic hepatitis? Y/ N/ Unsure

How much alcohol do you consume on average in a week? |__|

(0) 0ml (1) <100ml (2) <200ml (3) >=300ml

Do you smoke? Never/Former/ Current

How many cigarettes you do smoke on average in a day?

# Migration

Were you born in this village? No N; Yes Y; Don’t know D

Have you lived in this village all your life? |__| No N; Yes Y; Don’t know D

If No in the previous question ,then how long have you lived in this village? |__|

(1) less than 1 year (2) 1-5 years; (3) 6-10 years; (4) more than 10 years no data =999

Do you expect to stay in this village for next 5 years? No N; Yes Y; Don’t know D

Do you constantly reside in this village? No N; Yes Y; Don’t know D

If No how many (1) days/week (2) weeks/month (3) months/year do you reside?

**Pedigree data**

Relatives living in same village: and their relationship to the respondent

E.g: If the respondent's father is living in the village: enter Relationship="1", and his ID details

Relative No Relationship House ID Individ ID Age Name

1 |__| |__|__|__| |__|__| |__|__| _____________

2 |__| |__|__|__| |__|__| |__|__| _____________

3 |__| |__|__|__| |__|__| |__|__| _____________

4 |__| |__|__|__| |__|__| |__|__| _____________

5 |__| |__|__|__| |__|__| |__|__| _____________

6 |__| |__|__|__| |__|__| |__|__| _____________

7 |__| |__|__|__| |__|__| |__|__| _____________

8 |__| |__|__|__| |__|__| |__|__| _____________

9 |__| |__|__|__| |__|__| |__|__| _____________

10 |__| |__|__|__| |__|__| |__|__| _____________

(**Relationship**: 1=father; 2=mother; 3=son; 4=daughter; 5=brother; 6=sister)

**Water contact**

Frequency in **spring**?

0 None; 1 More than once a week; 2 Between weekly and monthly; 3 Once per month

Mode (1): 1 fishing; 2 herding bovines; 3 cutting grass; 4 washing clothes;

5 swimming; 6 walking; 7 washing hands; 8 farming; 9 other

Mode (2): 1 fishing; 2 herding bovines; 3 cutting grass; 4 washing clothes;

5 swimming; 6 walking; 7 washing hands; 8 farming; 9 other

Frequency in **summer**?

0 None; 1 More than once a week; 2 Between weekly and monthly; 3 Once per month

Mode (1): 1 fishing; 2 herding bovines; 3 cutting grass; 4 washing clothes;

5 swimming; 6 walking; 7 washing hands; 8 farming; 9 other

Mode (2): 1 fishing; 2 herding bovines; 3 cutting grass; 4 washing clothes;

5 swimming; 6 walking; 7 washing hands; 8 farming; 9 other

Frequency in **autumn**?

0 None; 1 More than once a week; 2 Between weekly and monthly; 3 Once per month

Mode (1): 1 fishing; 2 herding bovines; 3 cutting grass; 4 washing clothes;

5 swimming; 6 walking; 7 washing hands; 8 farming; 9 other

Mode (2): 1 fishing; 2 herding bovines; 3 cutting grass; 4 washing clothes;

5 swimming; 6 walking; 7 washing hands; 8 farming; 9 other

Did you have contact with flood water in last year?: |__| 1 often; 2 Occasionally; 3 none

In the next 5 years ,do you expect to have contact with water such as in lakes, rivers, streams, ponds, canals or ditches? No N; Yes Y; Don’t know D

Interviewer:_____________Date of interview |__|__|__|__|/|__|__|/|__|__| yyyy/mm/dd

#### FORM 2A

# Primary Human Stool Collection

Year:

| **Name** | **AID** | **NID** | **HID** | **PID** | **Stool 1** | **Date 1** | **Reason for no stool 1** | **Stool 2** | **Date 2** | **Reason for no stool 2** |
| --- | --- | --- | --- | --- | --- | --- | --- | --- | --- | --- |
|  |  |  |  |  |  |  |  |  |  |  |
|  |  |  |  |  |  |  |  |  |  |  |
|  |  |  |  |  |  |  |  |  |  |  |
|  |  |  |  |  |  |  |  |  |  |  |
|  |  |  |  |  |  |  |  |  |  |  |
|  |  |  |  |  |  |  |  |  |  |  |
|  |  |  |  |  |  |  |  |  |  |  |
|  |  |  |  |  |  |  |  |  |  |  |
|  |  |  |  |  |  |  |  |  |  |  |
|  |  |  |  |  |  |  |  |  |  |  |
|  |  |  |  |  |  |  |  |  |  |  |
|  |  |  |  |  |  |  |  |  |  |  |
|  |  |  |  |  |  |  |  |  |  |  |
|  |  |  |  |  |  |  |  |  |  |  |
|  |  |  |  |  |  |  |  |  |  |  |
|  |  |  |  |  |  |  |  |  |  |  |
|  |  |  |  |  |  |  |  |  |  |  |
|  |  |  |  |  |  |  |  |  |  |  |
|  |  |  |  |  |  |  |  |  |  |  |
|  |  |  |  |  |  |  |  |  |  |  |
|  |  |  |  |  |  |  |  |  |  |  |
|  |  |  |  |  |  |  |  |  |  |  |
|  |  |  |  |  |  |  |  |  |  |  |
|  |  |  |  |  |  |  |  |  |  |  |
|  |  |  |  |  |  |  |  |  |  |  |
|  |  |  |  |  |  |  |  |  |  |  |
|  |  |  |  |  |  |  |  |  |  |  |
|  |  |  |  |  |  |  |  |  |  |  |
|  |  |  |  |  |  |  |  |  |  |  |
|  |  |  |  |  |  |  |  |  |  |  |
|  |  |  |  |  |  |  |  |  |  |  |
|  |  |  |  |  |  |  |  |  |  |  |
|  |  |  |  |  |  |  |  |  |  |  |

**Investigator sign:**

**Date(yyyy/mm/dd):**

**FORM 2B Primary Human Stool Examination**

Year:

| **AID** | **NID** | **HID** | **PID** | **Slide Code**  **(from A to F)** | **Egg Count** | **Other helminths** |
| --- | --- | --- | --- | --- | --- | --- |
|  |  |  |  |  |  |  |
|  |  |  |  |  |  |  |
|  |  |  |  |  |  |  |
|  |  |  |  |  |  |  |
|  |  |  |  |  |  |  |
|  |  |  |  |  |  |  |
|  |  |  |  |  |  |  |
|  |  |  |  |  |  |  |
|  |  |  |  |  |  |  |
|  |  |  |  |  |  |  |
|  |  |  |  |  |  |  |
|  |  |  |  |  |  |  |
|  |  |  |  |  |  |  |
|  |  |  |  |  |  |  |
|  |  |  |  |  |  |  |
|  |  |  |  |  |  |  |
|  |  |  |  |  |  |  |
|  |  |  |  |  |  |  |
|  |  |  |  |  |  |  |
|  |  |  |  |  |  |  |
|  |  |  |  |  |  |  |
|  |  |  |  |  |  |  |
|  |  |  |  |  |  |  |
|  |  |  |  |  |  |  |
|  |  |  |  |  |  |  |
|  |  |  |  |  |  |  |
|  |  |  |  |  |  |  |
|  |  |  |  |  |  |  |
|  |  |  |  |  |  |  |
|  |  |  |  |  |  |  |
|  |  |  |  |  |  |  |
|  |  |  |  |  |  |  |
|  |  |  |  |  |  |  |
|  |  |  |  |  |  |  |
|  |  |  |  |  |  |  |
|  |  |  |  |  |  |  |
|  |  |  |  |  |  |  |
|  |  |  |  |  |  |  |

OTHER HELMINTHS: A= Ascaris, B=Trichuris, C=Hookworm, D=Other

Investigator(sign) :

Date:

**FORM 2C Primary Individuals For Treatment**

***Year:***

| **Name** | **AID** | **NID** | **HID** | **PID** | **TREATMENT? Y/N** | **REASON IF NO** |
| --- | --- | --- | --- | --- | --- | --- |
|  |  |  |  |  |  |  |
|  |  |  |  |  |  |  |
|  |  |  |  |  |  |  |
|  |  |  |  |  |  |  |
|  |  |  |  |  |  |  |
|  |  |  |  |  |  |  |
|  |  |  |  |  |  |  |
|  |  |  |  |  |  |  |
|  |  |  |  |  |  |  |
|  |  |  |  |  |  |  |
|  |  |  |  |  |  |  |
|  |  |  |  |  |  |  |
|  |  |  |  |  |  |  |
|  |  |  |  |  |  |  |
|  |  |  |  |  |  |  |
|  |  |  |  |  |  |  |
|  |  |  |  |  |  |  |
|  |  |  |  |  |  |  |
|  |  |  |  |  |  |  |
|  |  |  |  |  |  |  |
|  |  |  |  |  |  |  |
|  |  |  |  |  |  |  |
|  |  |  |  |  |  |  |
|  |  |  |  |  |  |  |
|  |  |  |  |  |  |  |
|  |  |  |  |  |  |  |
|  |  |  |  |  |  |  |
|  |  |  |  |  |  |  |
|  |  |  |  |  |  |  |
|  |  |  |  |  |  |  |
|  |  |  |  |  |  |  |
|  |  |  |  |  |  |  |
|  |  |  |  |  |  |  |
|  |  |  |  |  |  |  |
|  |  |  |  |  |  |  |
|  |  |  |  |  |  |  |

**REASON IF NO:**

**1= refused exam; 2= work outside area; 3=pregnant; 4=death; 5=other**

**Investigator(sign) :**

**Date:**

# FORM 2D

# Follow-Up Human Stool Collection

**Name**

**Administrative Village id □□□**

# Natural Village id □□

**House id □□□**

**Individual id □□□□ (0001 – nth)**

**Stool Number: ___(1 or 2)**

**Stool Specimen? □ (Y/N)**

**If Yes, Date collected stool: □□□□/□□/□□(yyyy/mm/dd)**

**If No, reason for no stool： □ (refused 1 not at home 2 sick 3 others 4)**

**FORM 2E**

**Follow-up Human Stool Examination:**

| **Date** | **ID** | **Slide** | **EPG** | **Other helminths** |
| --- | --- | --- | --- | --- |
|  |  |  |  |  |
|  |  |  |  |
|  |  |  |  |
|  |  |  |  |
|  |  |  |  |
|  |  |  |  |
|  |  |  |  |
|  |  |  |  |
|  |  |  |  |
|  |  |  |  |
|  |  |  |  |
|  |  |  |  |
|  |  |  |  |
|  |  |  |  |
|  |  |  |  |
|  |  |  |  |
|  |  |  |  |
|  |  |  |  |
|  |  |  |  |
|  |  |  |  |
|  |  |  |  |
|  |  |  |  |
|  |  |  |  |
|  |  |  |  |
|  |  |  |  |
|  |  |  |  |
|  |  |  |  |
|  |  |  |  |
|  |  |  |  |
|  |  |  |  |
|  |  |  |  |
|  |  |  |  |
|  |  |  |  |
|  |  |  |  |
|  |  |  |  |
|  |  |  |  |
|  |  |  |  |
|  |  |  |  |
|  |  |  |  |

**Investigator ID |__|__|**

OTHER HELMINTHS: A= Ascaris, B=Trichuris, C=Hookworm, D=Other

# FORM 3A

# Bovine Stool Collection

# YEAR:

| **Name of animal host** | **AID** | **NID** | **HID** | **BID** | **Type**  **cattle =1**  **buffalo 2** | **Age**  **(month)** | **Sex**  **F/M** | **Graze Location** | **Stool**  **Specimen? (Y/N)** | **Date for stool** | **Reason for no stool** | **migration** | | |  |
| --- | --- | --- | --- | --- | --- | --- | --- | --- | --- | --- | --- | --- | --- | --- | --- |
|  |  |  |  |  |  |  |  |  |  |  |  | **Animal ,Out or In!**  **(Leave/death= -1**  **New arrival= 0**  **Already present= 1**  **new born=2)** | **The date of**  **out or in?**  **(yyyy/mm/dd)** | **Where is the animal from?** | |
|  |  |  |  |  |  |  |  |  |  |  |  |  |  |  | |
|  |  |  |  |  |  |  |  |  |  |  |  |  |  |  | |
|  |  |  |  |  |  |  |  |  |  |  |  |  |  |  | |
|  |  |  |  |  |  |  |  |  |  |  |  |  |  |  | |
|  |  |  |  |  |  |  |  |  |  |  |  |  |  |  | |
|  |  |  |  |  |  |  |  |  |  |  |  |  |  |  | |
|  |  |  |  |  |  |  |  |  |  |  |  |  |  |  | |
|  |  |  |  |  |  |  |  |  |  |  |  |  |  |  | |
|  |  |  |  |  |  |  |  |  |  |  |  |  |  |  | |
|  |  |  |  |  |  |  |  |  |  |  |  |  |  |  | |
|  |  |  |  |  |  |  |  |  |  |  |  |  |  |  | |
|  |  |  |  |  |  |  |  |  |  |  |  |  |  |  | |
|  |  |  |  |  |  |  |  |  |  |  |  |  |  |  | |
|  |  |  |  |  |  |  |  |  |  |  |  |  |  |  | |
|  |  |  |  |  |  |  |  |  |  |  |  |  |  |  | |
|  |  |  |  |  |  |  |  |  |  |  |  |  |  |  | |
|  |  |  |  |  |  |  |  |  |  |  |  |  |  |  | |
|  |  |  |  |  |  |  |  |  |  |  |  |  |  |  | |
|  |  |  |  |  |  |  |  |  |  |  |  |  |  |  | |
|  |  |  |  |  |  |  |  |  |  |  |  |  |  |  | |

Investigator sign:

**Date(yyyy/mm/dd):**

**FORM 3C**  **Bovine Stool Examination**

Year:

| **AID** | **NID** | **HID** | **BID** | **MHT done?**  **( yes =Y;**  **no = N;**  **do not know = D)** | **Reason no MHT**  **(no stool=1**  **driedstoolspecimen= 2 other= 3 )** | **MHT (+/-)** | **Egg Count (if+)** |
| --- | --- | --- | --- | --- | --- | --- | --- |
|  |  |  |  |  |  |  |  |
|  |  |  |  |  |  |  |  |
|  |  |  |  |  |  |  |  |
|  |  |  |  |  |  |  |  |
|  |  |  |  |  |  |  |  |
|  |  |  |  |  |  |  |  |
|  |  |  |  |  |  |  |  |
|  |  |  |  |  |  |  |  |
|  |  |  |  |  |  |  |  |
|  |  |  |  |  |  |  |  |
|  |  |  |  |  |  |  |  |
|  |  |  |  |  |  |  |  |
|  |  |  |  |  |  |  |  |
|  |  |  |  |  |  |  |  |
|  |  |  |  |  |  |  |  |
|  |  |  |  |  |  |  |  |
|  |  |  |  |  |  |  |  |
|  |  |  |  |  |  |  |  |
|  |  |  |  |  |  |  |  |
|  |  |  |  |  |  |  |  |
|  |  |  |  |  |  |  |  |
|  |  |  |  |  |  |  |  |
|  |  |  |  |  |  |  |  |
|  |  |  |  |  |  |  |  |
|  |  |  |  |  |  |  |  |
|  |  |  |  |  |  |  |  |
|  |  |  |  |  |  |  |  |
|  |  |  |  |  |  |  |  |
|  |  |  |  |  |  |  |  |
|  |  |  |  |  |  |  |  |
|  |  |  |  |  |  |  |  |
|  |  |  |  |  |  |  |  |
|  |  |  |  |  |  |  |  |
|  |  |  |  |  |  |  |  |
|  |  |  |  |  |  |  |  |
|  |  |  |  |  |  |  |  |
|  |  |  |  |  |  |  |  |

**INVESTIGATOR(sign):**

**DATE:______________(yyyy/mm/dd)**

**FORM 3D Bovine Treatment**

**YEAR:**

| **Name of animal host** | **AID** | **NID** | **HID** | **BID** | **Dose of treatment** | **Reason for no treatment**  **refused exam= 1**  **work outside area=2 pregnant=3**  **death =4**  **other =5** |
| --- | --- | --- | --- | --- | --- | --- |
|  |  |  |  |  |  |  |
|  |  |  |  |  |  |  |
|  |  |  |  |  |  |  |
|  |  |  |  |  |  |  |
|  |  |  |  |  |  |  |
|  |  |  |  |  |  |  |
|  |  |  |  |  |  |  |
|  |  |  |  |  |  |  |
|  |  |  |  |  |  |  |
|  |  |  |  |  |  |  |
|  |  |  |  |  |  |  |
|  |  |  |  |  |  |  |
|  |  |  |  |  |  |  |
|  |  |  |  |  |  |  |
|  |  |  |  |  |  |  |
|  |  |  |  |  |  |  |
|  |  |  |  |  |  |  |
|  |  |  |  |  |  |  |
|  |  |  |  |  |  |  |
|  |  |  |  |  |  |  |
|  |  |  |  |  |  |  |
|  |  |  |  |  |  |  |
|  |  |  |  |  |  |  |
|  |  |  |  |  |  |  |
|  |  |  |  |  |  |  |
|  |  |  |  |  |  |  |
|  |  |  |  |  |  |  |
|  |  |  |  |  |  |  |
|  |  |  |  |  |  |  |
|  |  |  |  |  |  |  |
|  |  |  |  |  |  |  |
|  |  |  |  |  |  |  |
|  |  |  |  |  |  |  |
|  |  |  |  |  |  |  |
|  |  |  |  |  |  |  |
|  |  |  |  |  |  |  |
|  |  |  |  |  |  |  |
|  |  |  |  |  |  |  |
|  |  |  |  |  |  |  |

**Investigator sign:**

**Date(yyyy/mm/dd):**

**FORM 4**

**Human In-Migration Form**

## ID Information

Name___________________________________________________________

Province Administration village Natural village House Individual

|__| |__| |__|__| |__|__|__| |__|__|

Respondent for interview: |__| (1) Self; (2) Parent; (3) Child;

(4) Brother/sister; (5) Spouse; (6) Other

# Demographic Information

Sex |__| M/F Birth date |__|__|__|__|/|__|__|/|__|__| yyyy/mm/dd

Education |__| (1) Illiterate; (2) Semi-literate; (3) Primary school

(4) Junior School; (5) Senior School; (6) College

Years of education: |__|__|

Occupation: |__| (1) Farmer; (2) Fisherman; (3) Farmer + Fisherman; (4) Businessman; (5) Teacher; (6) Student; (7) Pre-School; (8) Other

# Schistosomiasis history

Have you ever had

Schistosomiasis? No N Yes Y Don’t know D

Advanced schistosomiasis? No N Yes Y Don’t know D

Acute schistosomiasis? No N Yes Y Don’t know D

Number of previous treatment for the schistosomiasis? |__||__| times

Have you had schistosomiasis in 2 years? No N Yes Y Don’t know D

In the last 2 weeks have you had:

Diarrhea? 0 No; 1 Often; 2 A few times; 3 Don’t know

Weakness? 0 No; 1 Often; 2 A few times; 3 Don’t know

Fever? 0 No; 1 Often; 2 A few times; 3 Don’t know

**Water contact**

Frequency in last Season?

0 None; 1 More than once a week; 2 Between weekly and monthly; 3 Once per month

Mode (1): 1 fishing; 2 herding bovines; 3 cutting grass; 4 washing clothes;

5 swimming; 6 walking; 7 washing hands; 8 farming; 9 other

Mode (2): 1 fishing; 2 herding bovines; 3 cutting grass; 4 washing clothes;

5 swimming; 6 walking; 7 washing hands; 8 farming; 9 other

Did you have contact with flood water in last year?: |__| 1 often; 2 Occasionally; 3 none

In the next 5 years ,do you expect to have contact with water such as in lakes, rivers, streams, ponds, canals or ditches? No N; Yes Y; Don’t know D

# Migration

Which village were you born in? ___________________

When did you come to this village? |__|__|__|__|/|__|__|/|__|__| yyyy/mm/dd

Why did you come to this village? ___________________

Do you expect to stay in this village for next 5 years? No N; Yes Y; Don’t know D

Do you constantly reside in this village? No N; Yes Y; Don’t know D

If No how many (1) days/week (2) weeks/month (3) months/year do you reside?

Interviewer:_____________Date of interview |__|__|__|__|/|__|__|/|__|__| yyyy/mm/dd

**FORM 5**

**Human Out-Migration Form**

## ID Information

Name___________________________________________________________

Province Administration village Natural village House Individual

|__| |__| |__|__| |__|__|__| |__|__|

Respondent for interview: |__| (1) Self; (2) Parent; (3) Child;

(4) Brother/sister; (5) Spouse; (6) Other

When are you leaving/when did you leave this village |__|__|__|__|/|__|__|/|__|__| yyyy/mm/dd

Why did you leave this village? ______________________________

Interviewer:_____________Date of interview |__|__|__|__|/|__|__|/|__|__| yyyy/mm/dd

**FORM 6**

### Water Contact Data Collection

The following information will be gathered for the current day (divided am and pm), and the previous day, for 8 randomly chosen days throughout the month (October) following the transmission season; i.e. a total of 32 measures. This enabled computation of an estimated minutes per day of water contact, per person.

| **Variable** | **Description** | **Coding** |
| --- | --- | --- |
| House ID | Household ID |  |
| ID | Individual identifier |  |
| NAME | Name of person |  |
| SEX | Sex of person | 1=Male  0=Female  9=Missing |
| AGE | Date of birth | Mm/dd/yy  Missing = 07/15/99 |
| WAYxyz | Mode of water contact  x=number of survey  y=day of contact  z=time of day of contact | 1=Fishing  2=Herding bovine  3=Cutting grass  4=Washing clothes  5=Swimming  6=Playing  7=Washing hands and walking  8=Others  9=Missing  Not recorded=missing  x=1-8  y: 1= today, 2= yesterday  z: 1= morning, 2= afternoon |
| POSxyz | Part of body immersed | 1=up to both ankles  2=up to both knees  3=up to buttocks  4=up to waist  5=up to neck  6=up to both wrists  7=up to both elbows  8=up to both shoulders |
| TIMExyz | Time of exposure | In minutes |
| SNAILxyz | Snails at contact site? | 1 = Yes  2 = No  0,3 = Do not know |
| PLACExyz | Contact sites | Site coding |
